# Virological suppression and clinical management in response to viremia in South African HIV treatment program: A multicenter cohort study

**DOI:** 10.1371/journal.pmed.1003037

**Published:** 2020-02-25

**Authors:** Lucas E. Hermans, Sergio Carmona, Monique Nijhuis, Hugo A. Tempelman, Douglas D. Richman, Michelle Moorhouse, Diederick E. Grobbee, Willem D. F. Venter, Annemarie M. J. Wensing

**Affiliations:** 1 Virology, Department of Medical Microbiology, University Medical Center Utrecht (UMCU), Utrecht, the Netherlands; 2 Wits Reproductive Health and HIV Institute (Wits RHI), University of the Witwatersrand, Johannesburg, South Africa; 3 Ndlovu Research Consortium, Elandsdoorn, South Africa; 4 Department of Molecular Medicine and Haematology, University of the Witwatersrand, Johannesburg, South Africa; 5 National Health Laboratory Service (NHLS), Johannesburg, South Africa; 6 University of the Witwatersrand, Johannesburg, South Africa; 7 Center for AIDS Research, University of California San Diego, United States of America; 8 VA San Diego Healthcare System, California, United States of America; 9 Clinical Epidemiology, University Medical Center Utrecht (UMCU), Utrecht, the Netherlands; 10 Julius Center for Health Sciences and Primary Care, Utrecht, the Netherlands; University of Southampton, UNITED KINGDOM

## Abstract

**Background:**

Uptake of antiretroviral treatment (ART) is expanding rapidly in low- and middle-income countries (LMIC). Monitoring of virological suppression is recommended at 6 months of treatment and annually thereafter. In case of confirmed virological failure, a switch to second-line ART is indicated. There is a paucity of data on virological suppression and clinical management of patients experiencing viremia in clinical practice in LMIC. We report a large-scale multicenter assessment of virological suppression over time and management of viremia under programmatic conditions.

**Methods and findings:**

Linked medical record and laboratory source data from adult patients on first-line ART at 52 South African centers between 1 January 2007 and 1 May 2018 were studied. Virological suppression, switch to second-line ART, death, and loss to follow-up were analyzed. Multistate models and Cox proportional hazard models were used to assess suppression over time and predictors of treatment outcomes. A total of 104,719 patients were included. Patients were predominantly female (67.6%). Median age was 35.7 years (interquartile range [IQR]: 29.9–43.0). In on-treatment analysis, suppression below 1,000 copies/mL was 89.0% at month 12 and 90.4% at month 72. Suppression below 50 copies/mL was 73.1% at month 12 and 77.5% at month 72. Intention-to-treat suppression was 75.0% and 64.3% below 1,000 and 50 copies/mL at month 72, respectively. Viremia occurred in 19.8% (20,766/104,719) of patients during a median follow-up of 152 (IQR: 61–265) weeks. Being male and below 35 years of age and having a CD4 count below 200 cells/μL prior to start of ART were risk factors for viremia. After detection of viremia, confirmatory testing took 29 weeks (IQR: 16–54). Viral resuppression to below 1,000 copies/mL without switch of ART occurred frequently (45.6%; 6,030/13,210) but was associated with renewed viral rebound and switch. Of patients with confirmed failure who remained in care, only 41.5% (1,872/4,510) were switched. The median time to switch was 68 weeks (IQR: 35–127), resulting in 12,325 person-years spent with a viral load above 1,000 copies/mL. Limitations of this study include potential missing data, which is in part addressed by the use of cross-matched laboratory source data, and the possibility of unmeasured confounding.

**Conclusions:**

In this study, 90% virological suppression below the threshold of 1,000 copies/mL was observed in on-treatment analysis. However, this target was not met at the 50-copies/mL threshold or in intention-to-treat analysis. Clinical management in response to viremia was profoundly delayed, prolonging the duration of viremia and potential for transmission. Diagnostic tools to establish the cause of viremia are urgently needed to accelerate clinical decision-making.

## Abstract 2

Lucas Hermans and co-authors study virological suppression in people with HIV receiving antiretroviral therapy during 2007-2018, in South Africa.

## Author summary

**Why was this study done?:**

- The global community has set ambitious targets for virological suppression during antiretroviral treatment (ART) for HIV infection, with the ultimate goal of halting the HIV epidemic.
- Data from clinical trials and cohorts show that these suppression rates are attainable. However, there is a paucity of data on actual suppression rates from large-scale treatment programs in low- and middle-income countries (LMIC), where the majority of HIV-infected patients reside.

**What did the researchers do and find?:**

- We studied virological suppression in a large multicenter observational cohort in LMIC reflecting clinical care under programmatic conditions of over 100,000 HIV-infected patients at 52 South African treatment sites.
- In this cohort, a virological suppression rate of 90% was attained below the 1,000-copies/mL HIV-DNA threshold. However, in intention-to-treat analysis, the virological suppression rate was substantially lower.
- The clinical management in response to viremia was delayed, resulting in a substantially prolonged duration of viremia at levels allowing for transmission.

**What do these findings mean?:**

- Our findings indicate that although ART is highly successful, viremia still occurs frequently in clinical practice.
- The delay in response to viremia contributes to a stable and large pool of potentially infectious patients.

## Introduction

The global community has adopted ambitious targets for rollout and success rates of antiretroviral treatment (ART) with the ultimate goal of halting the HIV epidemic. These 90-90-90 targets, set by the Joint United Nations Program on HIV/AIDS (UNAIDS), stipulate that suppression of the HIV-RNA load (viral load [VL]) should be achieved in 90% of HIV-infected patients on ART by 2020 [1]. Recently, this target was increased to 95% by 2030 [2].

Several cohorts in high-income countries (HICs) as well as recent clinical trials have reported virological suppression rates exceeding this ambitious target [3–5]. However, there is a paucity of virological suppression data from large representative cohorts in low- and middle-income countries (LMIC). Comparing suppression rates between different settings is complicated, as suppression is typically reported at a more lenient VL threshold of 1,000 copies/mL in LMIC [2,6]. As has previously been shown, this practice categorizes patients in whom viral replication is not controlled as being successfully treated [7,8]. The use of this threshold in reports of virological suppression may overestimate reported suppression rates in LMIC.

In addition, the frequency of VL monitoring during treatment varies globally. WHO guidelines, which are generally applied in LMIC, advise annual VL monitoring after attaining virological suppression [6], whereas in HICs, 3- to 4-monthly monitoring is advised, at least during the first years of treatment [9,10]. The use of a lower frequency of viral monitoring in LMIC may potentially delay clinical interventions after viral rebound and may result in prolonged episodes of viremia during ART, allowing for accumulation of resistance and onward transmission of (drug-resistant) HIV [11,12]. Given these risks, it is vital that clinical interventions as recommended by WHO guidelines, consisting of prompt confirmatory VL testing and switch to second-line treatment, are adhered to in order to maintain high overall rates of virological suppression. However, data on clinical management of patients experiencing viremia in LMIC are limited.

Large-scale assessment of VL data from programmatic clinical care in LMIC is urgently needed in order to assess virological suppression and clinical management of viremia. We report a longitudinal assessment of virological suppression and clinical management of viremia in a large South African multicenter cohort of patients receiving programmatic HIV treatment and monitoring.

## Methods

### Study design

We performed an analysis of routinely collected clinical and laboratory data in an open multicenter observational cohort of HIV-infected patients receiving ART at South African healthcare facilities. Data from this cohort have been reported previously [8]. Data analysis and reporting were performed in accordance with the RECORD statement (S1 Appendix) [13]. This study is the result of an exploratory analysis of virological suppression and follow-up after viremia without a prespecified analysis plan, which received ethical approval from the University of Witwatersrand Human Research Ethics Committee in Johannesburg, South Africa (Ref: M160762), and the Research Ethics Committee of the Faculty of Health Sciences, University of Pretoria, South Africa (Ref: 238/2016). Because of the observational nature of this study using anonymized data, individual informed consent was not required.

### Study population

Adult HIV-infected patients on ART attending 52 urban and rural public healthcare facilities across four South African provinces were included in the analysis. ART was provided in the framework of the South African national ART program, which provides treatment free of charge using WHO-aligned ART regimens and laboratory monitoring including VL assessment. First-line ART consisted of two nucleoside reverse transcriptase inhibitors (NRTIs) and a non-NRTI (NNRTI), and second-line ART consisted of two NRTIs and a ritonavir-boosted protease inhibitor (PI/r). Virological monitoring consisted of VL measurements at 6 and 12 months after initiation of ART and annual measurements thereafter in case of virological suppression. Virological failure is defined by guidelines as a VL of ≥1,000 copies/mL confirmed by a second measurement within 12 weeks. VL testing was performed on plasma samples using the Roche COBAS AmpliPrep/COBAS TaqMan HIV-1 Test, version 2.0, or Roche COBAS 8800 system (Roche, CA, USA) and by other assays in a minority of cases.

Cohort patients were included in this analysis if they met inclusion criteria consisting of prescription of NNRTI-based first-line ART and availability of at least one VL result performed at least 20 weeks after initiation of ART. In patients treated with insufficiently potent ART (i.e., monotherapy, dual therapy), the episode during which this was prescribed was censored from the analysis. Patients with unknown age and/or sex were excluded from the analysis.

### Data extraction and quality control

Medical record data were extracted from the standardized Three Integrated Electronic Registers (Tier.NET) electronic medical databases (WAMTechnology, Stellenbosch, South Africa). Anonymized database records were subjected to quality control, which included removal of duplicated or ambiguous data points. After quality control, all records from HIV-infected patients registered on ART at the participating facilities between start of large-scale rollout of ART in South Africa (1 January 2007) and date of data extraction (1 May 2018) were screened for inclusion as described above. To verify accuracy of electronic medical records, records of patients that received laboratory testing from the National Health Laboratory Services (NHLS) were matched to primary NHLS source data using a probabilistic matching algorithm. Additional information regarding sourcing and curation of data is available in the supplementary materials (S2 Appendix).

### Data analysis

The primary endpoint was viremia during ART, defined as any VL measurement ≥ 1,000 copies/mL during first-line ART, and performed at least 20 weeks after initiation of first-line ART. Secondary endpoints were confirmed virological failure (defined as two consecutive VL measurements ≥ 1,000 copies/mL), switch to second-line ART (defined as switch from NNRTI-containing to PI-containing ART after viremia), resuppression after viremia without switch to second-line ART, death, and loss to follow-up (LTFU). Patients were considered LTFU in case of a premature end of follow-up of at least 12 weeks before 1 May 2018 without confirmed death or transfer out.

Virological suppression during follow-up was evaluated using a multistate model into which all available VL results performed at least 20 weeks after initiation of first-line ART until end of follow-up or switch to second-line ART were entered as a repeated measure. The model allowed for transition between discrete virological states based on the most recent VL result. Virological states were defined as virological suppression (most recent VL below 50 copies/mL), low-level viremia (most recent VL between 51 and 999 copies/mL), and viremia (most recent VL ≥ 1,000 copies/mL). In on-treatment analysis, patients were right-censored after the last available VL result on first-line ART. In intention-to-treat analysis, patients were right-censored after the last available VL if they were still in care, had transferred out, or had deceased but not if they had become lost to follow-up. In this case, patients were regarded as lost to follow-up from 12 weeks after their last recorded clinic visit until date of data extraction. On-treatment and intention-to-treat results were reported as proportions and visualized using Aalen-Johansen curves in order to evaluate whether the UNAIDS-defined 90% virological suppression target was attained.

Mixed-effects Cox proportional hazards models were performed in order to assess baseline risk factors for the primary and secondary study outcomes. In a separate analysis of the risk of renewed rebound after a first episode of rebound followed by resuppression, patients were classified according to virological state, which was entered as a time-dependent covariate in which the patient is dynamically allocated to an exposure group based on the most recent VL result. Virological states were virological suppression below 50 copies/mL, low-level viremia (at least one VL result 51–999 copies/mL), viremia with subsequent resuppression below 50 copies/mL without switch of ART, or viremia with subsequent resuppression to low-level viremia (51–999 copies/mL) without switch of ART. All Cox proportional hazards models were adjusted for factors at start of ART that are known to affect treatment outcomes, i.e., sex, age, CD4-positive T-lymphocyte count (CD4 count), and ART regimen. Province of origin was entered as a random effect. Age and CD4 count variables were dichotomized at the approximate median value in all multivariable analyses. Patient records with missing data on one or more of the covariates were censored from the multivariable analysis. Patients who did not reach any of the study outcomes were right-censored after their last available VL result. Results were reported as hazard ratios (HRs) and adjusted HRs (aHRs) and 95% confidence intervals (95% CIs) and displayed as Kaplan-Meier curves. Validity of the proportional hazards assumption was evaluated using Schoenfeld residuals.

Sensitivity analysis was performed in order to assess the robustness of the medical record data. A comparative analysis of the primary study endpoint of viremia and virological suppression during follow-up was performed using results from medical record data and results from matched laboratory source data. In addition, stratified analyses of suppression by sex, CD4 count category, age category, and clinical setting were performed. A sensitivity analysis of Cox proportional hazards models was performed in which only patients with at least two VL results during treatment were entered.

## Results

### Patient characteristics

**Fig 1:**
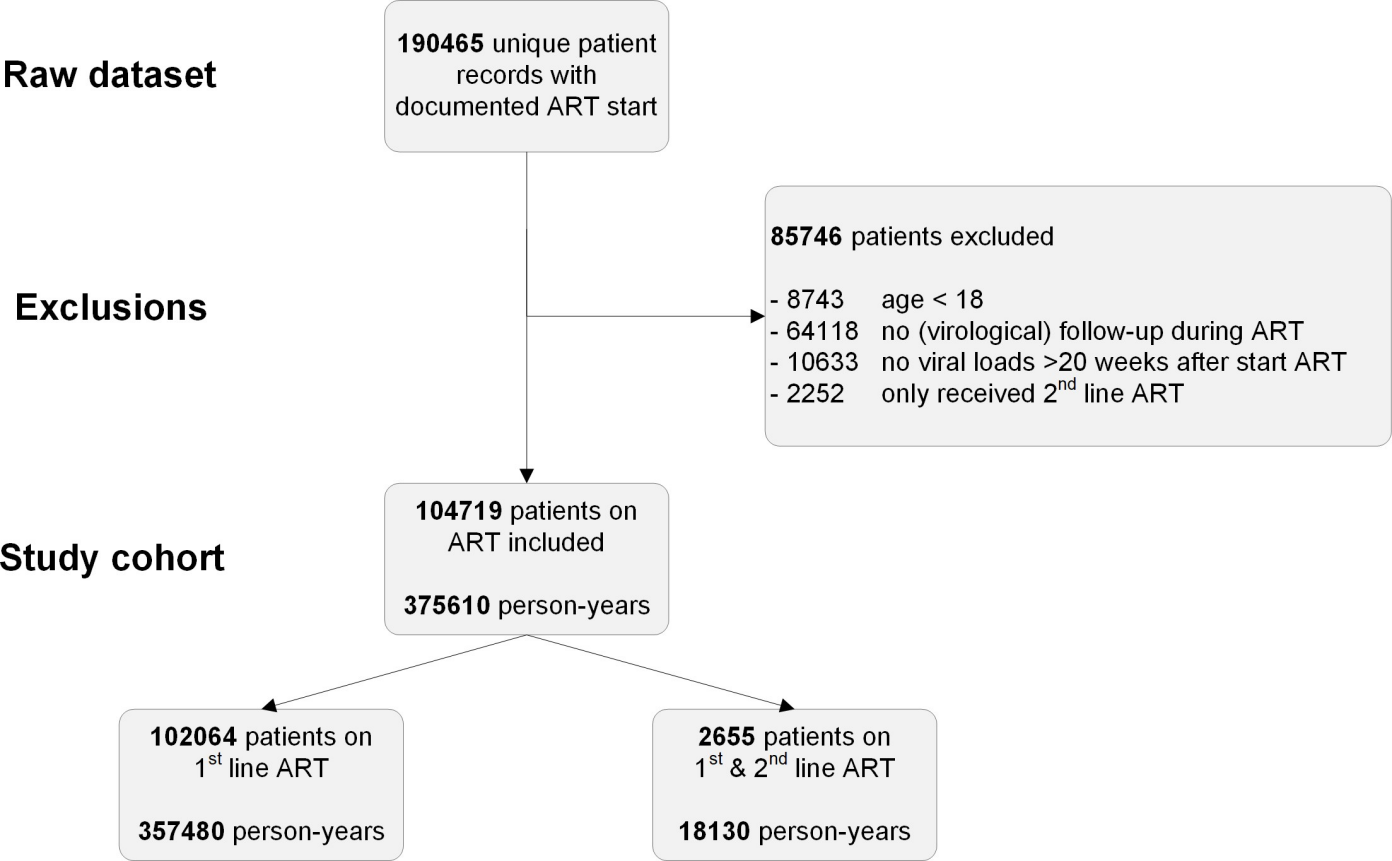
Patient inclusion. ART, antiretroviral treatment.

Data from 190,465 HIV-infected patients with recorded start of ART were screened for inclusion. Of screened patients, 8,743 were below 18 years of age at start of ART, and 2,252 patients exclusively received second-line ART during study follow-up. For 64,118 patients, no VL results were available during study follow-up, and in a further 10,633 patients, VL results were available but obtained prior to week 20 of ART. This resulted in the inclusion of 104,719 patients in the analysis (Fig 1). Median duration of follow-up on first-line ART was 152 weeks (interquartile range [IQR]: 61–265). Patients were predominantly female (67.6%). Median age was 35.7 years (IQR: 29.9–43.0). Most patients were treated with the NNRTI efavirenz (98.4%) and an NRTI backbone containing tenofovir (96.1%) (Table 1).

**Table 1:** Patient characteristics.

Characteristics		Overall	No viremia	Viremia > 1,000 c/mL
		(*n* = 104,719)	(*n* = 83,953)	(*n* = 20,766)
Sex	female	70,819 (67.6)	57 636 (68.7)	13,183 (63.5)
Age at start ART	years	35.7 [29.9–43.0]	35.8 [30.0–43.2]	35.1 [29.3–42.2]
CD4 at start ART	cells/μL	214 [115–333]	227 [124–344]	173 [87–280]
CD4 at start ART (cat)	<200 cells/μL	42,732 (46.6)	32,139 (43.8)	10,593 (58.3)
	200–500 cells/μL	40,196 (43.9)	33,579 (45.7)	6,617 (36.4)
	≥500 cells/μL	8,690 (9.5)	7,728 (10.5)	962 (5.3)
CD4 nadir during ART*	cells/μL	214 [112–340]	233 [124–358]	156 [73–262]
CD4 recovery during ART*	cells/μL	118 [0–315]	138 [0–331]	58 [0–234]
Viral loads during ART*	1	34,249 (32.7)	28,949 (34.5)	5,300 (25.5)
	2	25,613 (24.5)	21,066 (25.1)	4,547 (21.9)
	≥3	44,857 (42.8)	33,938 (40.4)	10,919 (52.6)
Follow-up on ART*	weeks	152 [61–265]	147 [57–263]	162 [79–280]
Follow-up on ART (cat)*	<1 years	18,549 (17.7)	15,719 (18.7)	2,830 (13.6)
	1–3 years	35,625 (34.0)	28,615 (34.1)	7,010 (33.8)
	≥3 years	50,545 (48.3)	39,619 (47.2)	10,926 (52.6)
Start year first-line ART	<2010	16,720 (16.0)	12,737 (15.2)	3,983 (19.2)
	2010–2013	30,874 (29.5)	23,622 (28.1)	7,252 (34.9)
	≥2013	57,125 (54.6)	47,594 (56.7)	9,531 (45.9)
NRTI exposure**	TDF and (FTC/3TC)	100,626 (96.1)	80,829 (96.3)	19,797 (95.3)
	ABC and 3TC	2,127 (2.0)	1,373 (1.6)	754 (3.6)
	AZT and 3TC	9,156 (8.7)	5,661 (6.7)	3,495 (16.8)
	d4t-containing	16,978 (16.2)	12,827 (15.3)	4,151 (20.0)
	ddI-containing	187 (0.2)	106 (0.1)	81 (0.4)
NNRTI exposure**	efavirenz	103,074 (98.4)	82,881 (98.7)	20,193 (97.2)
	nevirapine	8,920 (8.5)	6,579 (7.8)	2,341 (11.3)
Setting	Urban	44,517 (42.5)	38,785 (46.2)	5,732 (27.6)
	Urban-rural mixed	49,921 (47.7)	37,251 (44.4)	12,670 (61.0)
	Rural	10,281 (9.8)	7,917 (9.4)	2,364 (11.4)

Note: Data are *n* (%) or median [IQR]. Because of rounding, some percentages do not total 100%.

*Measured from start of first line of ART to last viral load during first-line ART.

**Measured as cumulative exposure.

†Univariate testing was performed using chi-squared tests for variables reported as percentages and using Mann-Whitney U tests for variables reported as medians.

Abbreviations: 3TC, lamivudine; ABC, abacavir; ART, antiretroviral treatment; AZT, zidovudine; c/mL, copies per milliliter; cat, category; CD4, CD4+ T-lymphocyte count; cells/μL, cells per microliter; d4t, stavudine; ddI, didanosine; FTC, emtricitabine; IQR, interquartile range; NRTI, nucleos(t)ide reverse transcriptase inhibitor; NNRTI, non-nucleoside reverse transcriptase inhibitor; TDF, tenofovir disoproxil fumarate

### Outcomes of ART

**Fig 2:**
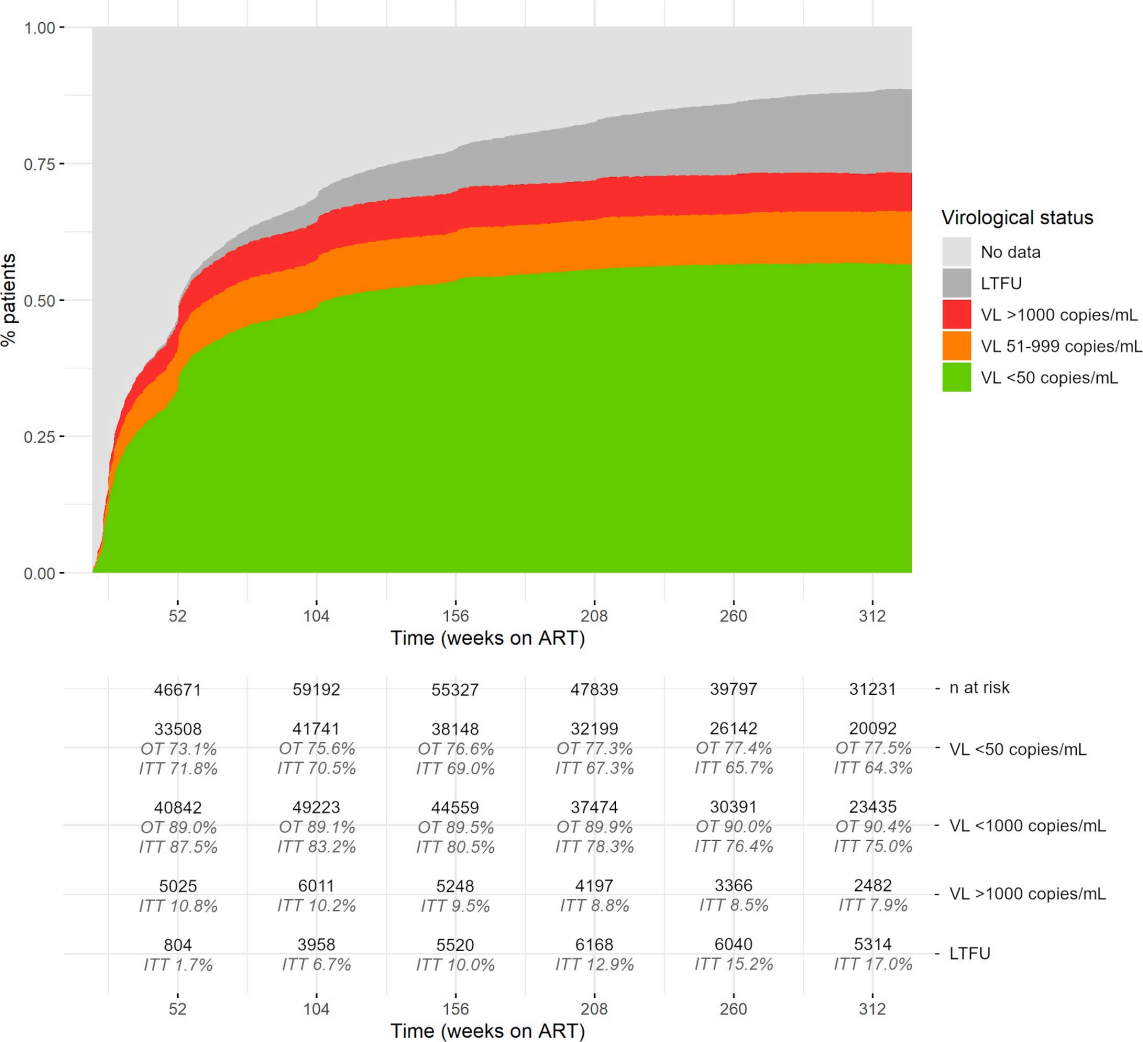
Multistate model of virological suppression during follow-up on first-line ART. Note: Multistate models of 104,719 patients on first-line ART. ART, antiretroviral treatment; ITT, intention-to-treat; LTFU, loss to follow-up; OT, on-treatment; VL, viral load.

The overall on-treatment virological suppression rate below 1,000 copies/mL was 89.0% at week 52 (1 year) and 90.4% at week 312 (6 years) of ART, whereas suppression below 50 copies/mL was 73.1% at 1 year and 77.5% at 6 years of ART (Fig 2). In intention-to-treat analysis, suppression below 1,000 copies/mL was 87.5% at 1 year and 75.0% at 6 years, whereas suppression below 50 copies/mL was 71.8% at 1 year to 64.3% at 6 years (Fig 2). Proportions with a VL between 51 and 999 copies/mL and a VL ≥ 1,000 copies/mL demonstrated a statistically significant downward trend over time (chi-squared test for trend in proportions: *p* < 0.001). The proportion of patients with no VL data after 6 years of follow-up was 3.8%.

During follow-up, 20,766 (19.8%) patients experienced viremia on first-line ART. In 6,018 (29.0%) of these patients, this occurred at the first VL measurement and during the first year of ART, suggesting an insufficient viral response rather than viral rebound. Overall, viremia occurred after a median duration of 92 weeks of first-line ART (IQR: 46–183). Death during follow-up occurred in 2.0% (2,105) of patients after a median 176 weeks (IQR: 108–277) of first-line ART. LTFU occurred in 17.6% (18,407) of patients after a median of 160 weeks (IQR: 95–255), and 12.6% (13,158) of patients transferred out.

In stratified analysis of virological suppression, the proportion of patients with a VL below 1,000 copies/mL at 6 years was 88.9% in male patients, 89.1% in patients below 35 years of age at start of ART, and 89.5% in patients with a CD4 count below 200 cells/μL at start of ART (S3 Appendix). Each of these characteristics was independently associated with an increased hazard for viremia. The aHR for viremia was 1.39 (95% CI 1.35–1.43, *p* < 0.001) for male patients, 0.91 (95% CI 0.90–0.92, *p* < 0.001) per 5-year age increment, and 0.95 (95% CI 0.95–0.96, *p* < 0.001) per 50-cells/μL CD4 count increment. Increased hazards of switch to second-line ART were also observed for these risk groups (Table 2). Male sex was also associated with an increased hazard of LTFU (aHR 1.44, 95% CI 1.40–1.49, *p* < 0.001) and death (aHR 1.76, 95% CI 1.61–1.94, *p* < 0.001). Higher age was associated with a decreased risk of LTFU (aHR 0.90, 95% CI 0.90–0.91, *p* < 0.001) and an increased risk of death (aHR 1.16, 95% CI 1.14–1.19, *p* < 0.001), whereas higher CD4 count was associated with an increased risk of LTFU (aHR 1.02, 95% CI 1.01–1.02, *p* < 0.001) and a decreased risk of death (aHR 0.92, 95% CI 0.90–0.94, *p* < 0.001) (Table 3).

**Table 2:** Mixed-effects Cox proportional hazards analysis—Viremia and switch to second-line ART.

	Patients on first-line ART (*n* = 91,618)					
Outcome	Viremia > 1,000 copies/mL 19.8% (*n* = 18,172)			Switch to second-line ART 2.7% (*n* = 2,451)		
	% with outcome	HR	aHR	% with outcome	HR	aHR
Sex: male	22.4% (6,743/30,101)	1.29*** [1.25–1.33]	1.39*** [1.35–1.43]	3.0% (896/30,101)	1.25*** [1.15–1.36]	1.15*** [1.05–1.25]
Age at start ART	NA	0.95*** [0.94–0.96]	0.91*** [0.90–0.92]	NA	0.95*** [0.93–0.97]	0.92*** [0.90–0.94]
CD4 at start ART	NA	0.96*** [0.95–0.96]	0.95*** [0.95–0.96]	NA	0.79*** [0.77–0.80]	0.82*** [0.80–0.83]

Note: Analysis for patients on first-line ART with available data on all variables (*n* = 91,618). Because of missing data on CD4 at start ART, 13,101 out of 104,719 patients were not entered in the analysis. Results are displayed as the prevalence of the outcome for each variable and as HR/aHR [95% confidence intervals] of the outcome. Models were adjusted for ART regimen. Province was entered as a random effect. Age was assessed in increments of 5 years. CD4 count was assessed in increments of 50 cells/μL.

***p < 0.001.

Abbreviations: aHR, adjusted HR; ART, antiretroviral treatment; CD4, CD4+ T-lymphocyte count; cells/μL, cells per microliter; HR, hazard ratio; NA, not available

**Table 3:** Mixed-effects Cox proportional hazards analysis—Loss to follow-up and death.

	Patients on first-line ART (*n* = 91,618)					
Outcome	Loss to follow-up 17.6% (*n* = 16,148)			Death 2.0% (*n* = 1,855)		
	% with outcome	HR	aHR	% with outcome	HR	aHR
Sex: male	20.2% (6,090/30,101)	1.31*** [1.27–1.35]	1.44*** [1.40–1.49]	3.0% (899/30,101)	1.97*** [1.80–2.16]	1.76*** [1.61–1.94]
Age at start ART	NA	0.92*** [0.91–0.93]	0.90*** [0.90–0.91]	NA	1.25*** [1.23–1.28]	1.16*** [1.14–1.19]
CD4 at start ART	NA	1.02*** [1.01–1.02]	1.02*** [1.01–1.02]	NA	0.91*** [0.89–0.92]	0.92*** [0.90–0.94]

Note: Analysis for patients on first-line ART with available data on all variables (*n* = 91,618). Because of missing data on CD4 at start ART, 13,101 out of 104,719 patients were not entered in the analysis. Results are displayed as the prevalence of the outcome for each variable and as HR/aHR [95% confidence intervals] of the outcome. Models were adjusted for ART regimen. Province was entered as a random effect. Age was assessed in increments of 5 years. CD4 count was assessed in increments of 50 cells/μL.

***p < 0.001.

Abbreviations: aHR, adjusted HR; ART, antiretroviral treatment; CD4, CD4+ T-lymphocyte count; cells/μL, cells per microliter; HR, hazard ratio; NA, not available

Virological suppression below 1,000 copies/mL varied between geographical settings, from 94.6% in urban settings to 88.0% in rural and urban-rural mixed settings at 72 months in on-treatment analysis (S4 Appendix). Sensitivity analysis of virological suppression using matched laboratory source data resulted in highly similar suppression figures, indicating high robustness of the main analysis (S5 Appendix).

### Resuppression on first-line ART

Of patients with viremia, subsequent virological follow-up was available in 13,210 patients. In these patients, resuppression below 1,000 copies/mL at the next VL measurement without switch of ART was attained in 45.6% (6,030/13,210), whereas virological failure was confirmed in 54.4% (7,180/13,210). After confirmation of virological failure, 19.9% (1,431/7,180) subsequently achieved resuppression without switch of ART. However, resuppression was less profound in these patients, and low-level viremia (51–999 copies/mL) remained present in 46.3% (662/1,431), compared to 25.9% (1,559/6,030) of patients who resuppressed directly after one viremic episode (odds ratio 2.47, 95% CI 2.19–2.78, *p* < 0.001). In patients who experienced viremia, risk factors for viremia (male sex, lower age, and lower CD4 count) also increased the likelihood of confirmation of failure and decreased the likelihood of achieving resuppression (S6 Appendix).

**Fig 3:**
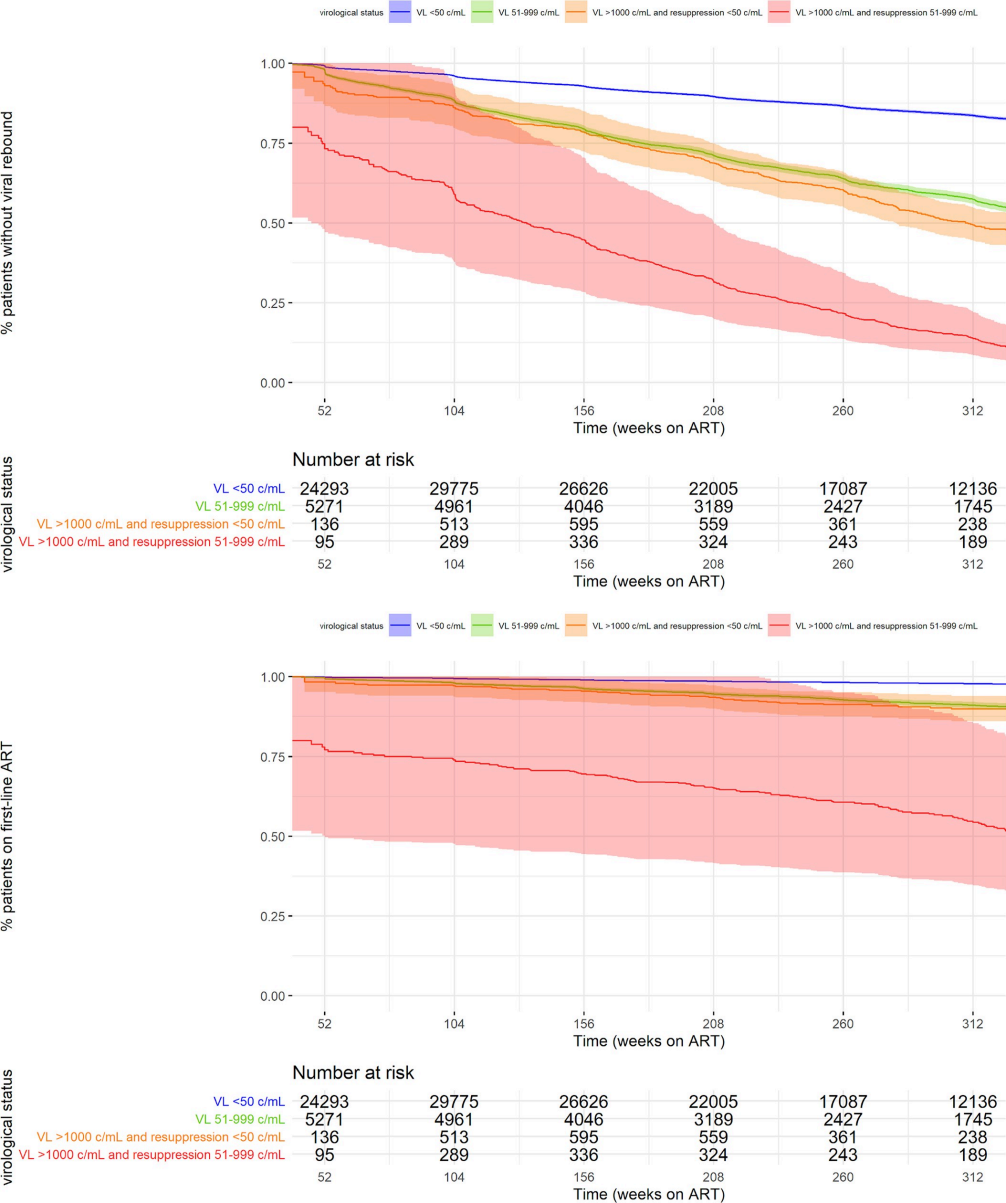
Mixed-models interval survival analysis—Viral rebound and switch to second-line ART. Note: Extended Kaplan-Meier estimators based on interval Cox proportional hazard analysis of 57,006 patients on first-line ART. Of 104,719 patients, exclusion was based on missing baseline CD4 count in 13,101, unavailability of more than one VL during first-line ART follow-up in 29,365, and initial virological failure without having more than one recorded VL below 1,000 c/mL in 5,247 cases. VL was entered as a time-dependent covariate. Numbers represent the amount of patients in the given virological state at a given time point. ART, antiretroviral treatment; c/mL, copies per milliliter; ITT, intention-to-treat; LTFU, loss to follow-up; OT, on-treatment; VL, viral load.

Patients who experienced resuppression remained at increased risk of renewed viral rebound. Subsequent rebound was observed in 13.0% (305/2,345) cases of resuppression to below 50 copies/mL and in 34.1% (568/1,665) of cases of resuppression to low-level viremia (51–999 copies/mL). Compared to sustained virological suppression below 50 copies/mL, the aHR for renewed rebound was 3.16 (95% CI 2.82–3.53, *p* < 0.001) after resuppression to below 50 copies/mL and 8.91 (95% CI 8.19–9.69, *p* < 0.001) after resuppression to low-level viremia. The hazard of switch to second-line ART was also significantly increased for both groups (Table 4, Fig 3).

**Table 4:** Mixed-models interval Cox proportional hazard analysis—Viral rebound and switch.

		Patients on first-line ART (*n* = 57,006; 147,153 VL intervals)					
Outcome		Viral rebound > 1,000 c/mL (*n* = 7,451 events)			Switch to second-line ART (*n* = 957 events)		
		% with outcome	HR	aHR	% with outcome	HR	aHR
VL history	Suppression (<50 c/mL)	3.5% (4,273/121,487)	Ref	Ref	0.4% (479/121,487)	Ref	Ref
	Low-level viremia (51–999 c/mL)	10.1% (2,177/21,656)	3.19*** [3.03–3.36]	2.98*** [2.83–3.13]	1.5% (326/21,656)	4.21*** [3.66–4.85]	4.10*** [3.55–4.72]
	Previous viremia and resuppression (<50 c/mL)	13.0% (305/2,345)	3.66*** [3.26–4.11]	3.16*** [2.82–3.53]	1.8% (42/2,345)	4.58*** [3.34–6.28]	4.87*** [3.55–6.66]
	Previous viremia and resuppression (51–999 c/mL)	34.1% (568/1,665)	10.91*** [9.99–11.91]	8.91*** [8.19–9.69]	6.6% (110/1,665)	19.23*** [15.62–23.68]	17.31*** [13.94–21.50]

Note: Interval survival analysis for patients on first-line ART with available data on all variables (*n* = 57,006). Of 104,719 patients, exclusion was based on missing baseline CD4 count in 13,101, unavailability of more than one VL during first-line ART follow-up in 29,365, and initial virological failure without having more than one recorded VL below 1,000 c/mL in 5,247 cases. VL was entered as a time-dependent covariate; 147,153 follow-up intervals between two VL measurements were entered in the analysis. The VL covariates reflect the VL status at the start of the interval; the outcome reflects the event at the end of the interval. Results are displayed as the prevalence per interval of the outcome for each variable and as HR/aHR [95% CI] of the outcome. Switch to second-line ART was defined as a switch from NNRTI-based to PI-based ART after at least one VL result > 1,000 c/mL. Cohort was entered as a random effect. Analysis was corrected for sex, age at start ART, CD4+ T-lymphocyte count at start ART, and ART with tenofovir, efavirenz, and lamivudine/emtricitabine versus other. Age was assessed in increments of 5 years. CD4 count was assessed in increments of 50 cells/μL. Low-level viremia was defined as one or more VL result 51–999 c/mL during ART.

***p < 0.001.

Abbreviations: aHR, adjusted HR; ART, antiretroviral treatment; c/mL, copies per milliliter; HR, hazard ratio; NNRTI, non-nucleoside reverse transcriptase inhibitor; PI, protease inhibitor; Ref, reference level; VL, viral load

### Confirmation of virological failure and switch to second-line ART

**Fig 4:**
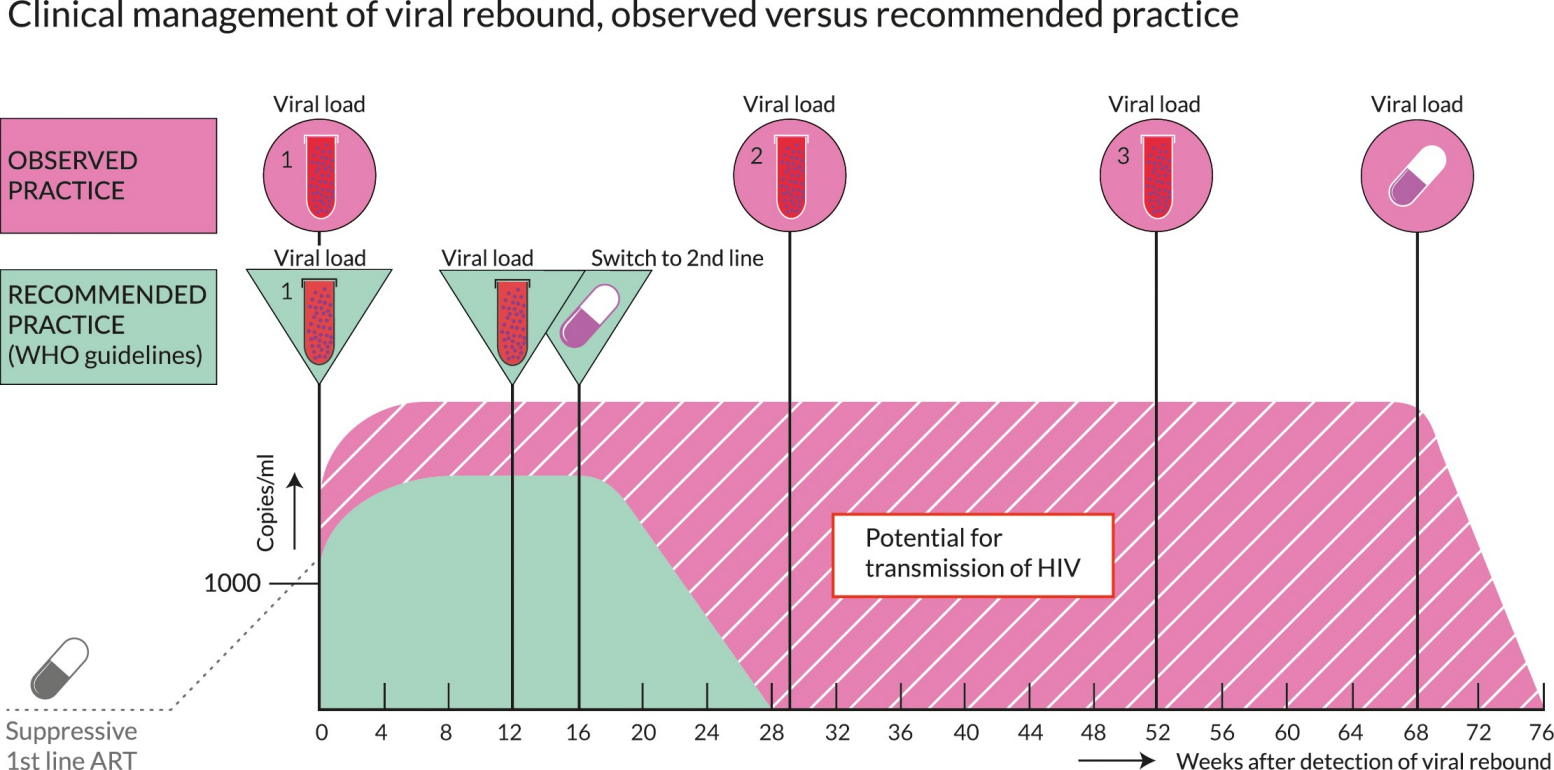
Clinical management of viremia—Observed versus recommended practice.

In patients with confirmed virological failure, confirmatory VL testing was generally not performed within the WHO-recommended timeframe of 12 weeks after detection of rebound. The median time between first detection of viremia and confirmation of virological failure was 29 weeks (IQR: 16–54) and varied between a minimum median of 17 weeks (IQR: 8–34) and a maximum median of 35 weeks (IQR: 21–61) between settings (S7 Appendix). Of patients who remained in care after confirmation of failure, only 41.5% (1,872/4,510) were switched to second-line ART during study follow-up. Of patients with follow-up after confirmation of virological failure but without documented switch to second-line ART, 5.8% (385/6,800) died, 19.3% (1,362/7,180) became lost to follow-up, and 8.1% (543/7,180) transferred out. Switching to second-line ART after confirmation of failure was generally not performed within recommended timeframes. The median time between first detection of viremia and switch was 68 weeks (IQR: 35–127). Median time to switch was prolonged in all settings, ranging from a minimum of 48 weeks (IQR: 25–87) to a maximum of 96 weeks (IQR: 56–150) (S7 Appendix). An average of 3.1 VL results above 1,000 copies/mL were available during this period, indicating that on average, at least one additional VL was performed after confirmed failure prior to switching to second-line ART (Fig 4). The average number of VL results during failure varied from 2.7 to 3.4 (S7 Appendix). The 2,638 patients with confirmed virological failure who were still in care but did not switch to second-line ART spent a median of 91 weeks (IQR: 50–154) with viremia above 1,000 copies/mL. In these patients, an average of 3.3 VL results above 1,000 copies/mL were present, varying from 2.9 to 3.5 between settings (S7 Appendix). In total, patients with confirmed virological failure spent 12,325 person-years with viremia above 1,000 copies/mL. Sensitivity analysis with matched laboratory source data revealed highly similar times from detection of rebound to confirmation and switch (S5 Appendix).

## Discussion

In this study, we demonstrate sustained high rates of virological suppression over time in South African patients on first-line ART in urban and rural settings. An on-treatment virological suppression rate of 90% was observed, with an intention-to-treat suppression rate of 75% after several years of follow-up. These results reflect the significant achievements of large-scale rollout of treatment, which has resulted in considerable health benefits and increased life expectancy [14,15].

The 90% target was only attained at the WHO-defined VL threshold of 1,000 copies/mL. There is a paucity of scientific evidence to support the use of this threshold. Previously, we and others have shown that viremia below this threshold during ART confers considerable risks of poor treatment outcomes and selection of drug resistance [7,8,16,17]. When a threshold of 50 copies/mL, as advised by several guidelines and adopted in clinical trials [18], was applied to define virological suppression, the suppression rate in this cohort dropped to 80%. This difference is profound, particularly as recent analyses of some European cohorts have reported on-treatment suppression rates around 95% at the 50-copies/mL threshold [4,5]. Stringent monitoring, individualized selection of treatment, and the availability of newer drug classes such as integrase inhibitors in these settings may explain the observed difference.

Intention-to-treat virological suppression below 1,000 copies/mL decreased to 75% after 6 years of follow-up. The observed differences between on-treatment and intention-to-treat suppression rates is mainly due to LTFU, which amounted to 17% after 6 years. Although this rate is much lower than in previous reports of LTFU across LMIC [19], it still implies that 90% virological suppression below 1,000 copies/mL is not attained if all patients who started ART are taken into account. It has been shown that although a small proportion of patients will reinitiate ART elsewhere, over half of patients who become lost to follow-up are either deceased or have discontinued treatment altogether [20].

Over 20% of patients in this cohort experienced viremia during follow-up. Young adults and men, as well as patients with poor baseline immunological status, were at increased risk of viremia, as has been observed in several African countries [21,22]. We demonstrate that in these subgroups, the 90% target for virological suppression was not attained even after years of treatment, which is likely mediated through suboptimal adherence. The high rate of treatment failure in these subgroups poses a serious concern, as it increases the risk of poor health outcomes [23,24]. The detection of baseline predictors for treatment response offers an opportunity for targeted interventions and increased vigilance based on risk profiles, which may improve health outcomes.

After viremia, a substantial number of patients achieved resuppression in absence of switching to second-line ART. This is in line with previous data from South Africa and other LMIC [25,26]. The long duration of follow-up in our cohort enabled us to demonstrate that these patients remained at increased risk for renewed viral rebound, particularly when low-level viremia remained present after resuppression.

Confirmatory VL testing after detection of viremia was profoundly delayed. This delay may be due to a variety of provider-driven, patient-related, or health infrastructural factors such as patient nonattendance [27,28]. In contrast, it has been suggested that subsequent delay between confirmation and switching to second-line ART is more likely provider-driven [29]. Our analysis shows that additional VL measurements were performed between confirmation and switch in most cases, confirming that delay cannot be solely explained by nonattendance. The apparent reluctance in clinical practice to switch to second-line ART results in a long duration of viremia of more than 1 year, on average, in patients with confirmed failure. This contributes to a stable and large pool of long-term infectious patients in the treated population. Efforts to minimize this pool are especially relevant, as the proposed 90-90-90 targets of UNAIDS may not be sufficient to curb the epidemic [30].

Lack of access to diagnostic tools that give insight in the reasons of viremia leaves healthcare practitioners ill-equipped to improve the current situation. If viremia is solely due to nonadherence, adherence interventions are indicated to prevent unnecessary switches to second-line ART [11,31]. However, if resistance has already been selected, a rapid switch can prevent accumulation of drug resistance and loss of therapeutic options [11]. Objective adherence monitoring by drug-level assessment and/or drug-resistance testing in patients with viremia may empower healthcare practitioners to swiftly respond to viremia and make informed switches to second-line ART.

The planned introduction of the integrase strand-transfer inhibitor dolutegravir in LMIC, driven by the increasing presence of drug-resistant viral strains in the population, may increase overall virological suppression rates but does little to allay concerns regarding management of viremia. As no drug is impervious to resistance, the introduction of a new drug class should be accompanied by expansion of monitoring tools to understand viremia, especially in the context of high levels of drug resistance to the NRTI backbone [32,33]. Understanding viremia and adequate subsequent clinical intervention has the potential to raise virological suppression in the population to levels required to finally curb the epidemic.

Reported virological suppression rates in LMIC vary widely [34]. The majority of available data originate from single-center studies performed at specialized clinics and hospitals [35–40]. Although the results of these studies are important, they are likely not representative of clinical care in large-scale treatment programs. Recently, some efforts to characterize suppression in larger populations of patients, using clinical database systems and population-based surveys, have been undertaken, but these remain of relatively modest size and scope [41–45]. Centralized laboratory databases have enabled cross-sectional analyses of VL data at a much larger scale and can be used to infer some clinical variables such as treatment start date and between-clinic transfer [21,46–48]. However, detailed assessment of ART outcomes in standard clinical care in LMIC treatment programs requires analysis of clinical data from these treatment programs. We used a well-curated medical records database as a primary data source and ensured validity of results by comparing them to cross-referenced records from a laboratory database. This analysis was designed to give an inclusive overview of viral suppression during first-line ART in this large cohort. The application of minimal inclusion and exclusion criteria was performed to minimize potential selection bias.

Limitations of this study include potential missing clinical and laboratory data. Missing data may originate from lack of adequate capturing but also from patient movement between clinics. Despite these limitations, cross-referencing of medical record data to laboratory source data demonstrated the robustness of the study findings. Although care was taken to adjust associations for known confounders, residual confounding in the multivariable analyses due to unmeasured covariates that cannot be adjusted for is an inherent risk to observational studies and may have affected the results. A degree of survival bias can be expected in longitudinal analysis of virological suppression, as patients who reach adverse treatment outcomes such as switch to second-line ART or LTFU are commonly censored from these analyses. We have sought to overcome this important limitation by performing an intention-to-treat analysis in which patients were not censored after switching to second-line ART and contributed follow-up time in case of LTFU. However, lack of data on patient outcomes after LTFU renders us unable to account for any subsequent outcomes of treatment, such as reengagement into care elsewhere and death. Although of considerable scale, the findings from this study may not be translatable to other LMIC. Despite these limitations, this analysis, covering a large number of urban and rural South African treatment centers, allows for unique insights into treatment outcomes in current clinical practice under programmatic conditions in LMIC.

In summary, high virological suppression rates were attained in the South African treatment program. However, one in five patients did not partake in this success. In these patients, clinical follow-up of VL results was delayed, potentially because of anticipation of resuppression and fear of unnecessary switches to second-line ART. The presence of a large subset of long-term infectious patients within the treated population poses a risk to the health of these individuals and impedes efforts to control the epidemic. Diagnostic tools to establish the cause of viremia are urgently required to perform targeted adherence interventions and informed timely switches to second-line ART.

## Supporting information

S1 Appendix RECORD checklist. (DOCX)

S2 Appendix Data sourcing, selection, and curation. (DOCX)

S3 Appendix Virological suppression stratified by risk factors for viremia. (DOCX)

S4 Appendix Virological suppression by setting. (DOCX)

S5 Appendix Virological suppression and follow-up after viremia by data source. (DOCX)

S6 Appendix Cox proportional hazard analysis. (DOCX)

S7 Appendix Follow-up after detection of viremia by setting. (DOCX)

## Abbreviations

95% CI: 95% confidence interval
ABC: abacavir
aHR: adjusted HR
ART: antiretroviral treatment
AZT: zidovudine
d4t: stavudine
ddI: didanosine
HIC: high-income country
HR: hazard ratio
IQR: interquartile range
LMIC: low- and middle-income countries
LTFU: loss to follow-up
NA: not available
NHLS: National Health Laboratory Services
NNRTI: non-NRTI
NRTI: nucleoside reverse transcriptase inhibitor
PI/r: ritonavir-boosted protease inhibitor
Ref: reference level
TDF: tenofovir disoproxil fumarate
Tier.NET: Three Integrated Electronic Registers
UNAIDS: Joint United Nations Program on HIV/AIDS
VL: viral load.
