# Supplementary material for: Virological suppression and clinical management in response to viremia in South African HIV treatment program: A multicenter cohort study
Source: PLoS Med. 2020 Feb 25;17(2):e1003037. doi: 10.1371/journal.pmed.1003037 (PMC7041795; doi:10.1371/journal.pmed.1003037)
Supplement: S1 Appendix — (DOCX) [file pmed.1003037.s001.docx]

## RECORD Checklist

|  | **Item No.** | **STROBE items** | **Location in manuscript where items are reported** | **RECORD items** | **Location in manuscript where items are reported** |
| --- | --- | --- | --- | --- | --- |
| **Title and abstract** | | | | | |
|  | 1 | (a) Indicate the study’s design with a commonly used term in the title or the abstract (b) Provide in the abstract an informative and balanced summary of what was done and what was found | *(a) Study design indicated in study title* | RECORD 1.1: The type of data used should be specified in the title or abstract. When possible, the name of the databases used should be included.  RECORD 1.2: If applicable, the geographic region and timeframe within which the study took place should be reported in the title or abstract.  RECORD 1.3: If linkage between databases was conducted for the study, this should be clearly stated in the title or abstract. | *(1.1) The type of data is indicated in the abstract Methods.*  *(1.2) geographic region is reported in title, timeframe is reported in the abstract Methods.*  *(1.3) Database linkage is reported in the abstract Methods.* |
| **Introduction** | | | | | |
| Background rationale | 2 | Explain the scientific background and rationale for the investigation being reported | *Introduction (paragraph 1-4)* |  |  |
| Objectives | 3 | State specific objectives, including any prespecified hypotheses | *Introduction (paragraph 4)* |  |  |
| **Methods** | | | | | |
| Study Design | 4 | Present key elements of study design early in the paper | *Methods, subsection “study design”* |  |  |
| Setting | 5 | Describe the setting, locations, and relevant dates, including periods of recruitment, exposure, follow-up, and data collection | *Methods, subsection “study population” and “data extraction and quality control”* |  |  |
| Participants | 6 | *(a) Cohort study* - Give the eligibility criteria, and the sources and methods of selection of participants. Describe methods of follow-up  *Case-control study* - Give the eligibility criteria, and the sources and methods of case ascertainment and control selection. Give the rationale for the choice of cases and controls  *Cross-sectional study* - Give the eligibility criteria, and the sources and methods of selection of participants  *(b) Cohort study* - For matched studies, give matching criteria and number of exposed and unexposed  *Case-control study* - For matched studies, give matching criteria and the number of controls per case | *(a – Cohort study) Methods, subsection “study population”* | RECORD 6.1: The methods of study population selection (such as codes or algorithms used to identify subjects) should be listed in detail. If this is not possible, an explanation should be provided.  RECORD 6.2: Any validation studies of the codes or algorithms used to select the population should be referenced. If validation was conducted for this study and not published elsewhere, detailed methods and results should be provided.  RECORD 6.3: If the study involved linkage of databases, consider use of a flow diagram or other graphical display to demonstrate the data linkage process, including the number of individuals with linked data at each stage. | *(6.1) Methods, subsection “study population”*  *(6.2) Previous work with this dataset has been referenced in Methods, subsection “study design”.*  *(6.3) The matching algorithm was described in supplementary materials (S6 Appendix)* |
| Variables | 7 | Clearly define all outcomes, exposures, predictors, potential confounders, and effect modifiers. Give diagnostic criteria, if applicable. | *Methods, subsection “data analysis”* | RECORD 7.1: A complete list of codes and algorithms used to classify exposures, outcomes, confounders, and effect modifiers should be provided. If these cannot be reported, an explanation should be provided. | *(7.1) The handling and definition of variables is described in detail in Methods, subsection “data analysis”* |
| Data sources/ measurement | 8 | For each variable of interest, give sources of data and details of methods of assessment (measurement).  Describe comparability of assessment methods if there is more than one group | *Methods, subsection “data analysis”* |  |  |
| Bias | 9 | Describe any efforts to address potential sources of bias | *Methods, subsection “data analysis”* |  |  |
| Study size | 10 | Explain how the study size was arrived at | *Methods, subsection “data extraction and quality control”* |  |  |
| Quantitative variables | 11 | Explain how quantitative variables were handled in the analyses. If applicable, describe which groupings were chosen, and why | *Methods, subsection “data analysis”* |  |  |
| Statistical methods | 12 | (a) Describe all statistical methods, including those used to control for confounding  (b) Describe any methods used to examine subgroups and interactions  (c) Explain how missing data were addressed  (d) *Cohort study* - If applicable, explain how loss to follow-up was addressed  *Case-control study* - If applicable, explain how matching of cases and controls was addressed  *Cross-sectional study* - If applicable, describe analytical methods taking account of sampling strategy  (e) Describe any sensitivity analyses | *Methods, subsection “data analysis”* |  |  |
| Data access and cleaning methods |  | .. |  | RECORD 12.1: Authors should describe the extent to which the investigators had access to the database population used to create the study population.  RECORD 12.2: Authors should provide information on the data cleaning methods used in the study. | *This is reported in the supplementary materials (S6 Appendix)* |
| Linkage |  | .. |  | RECORD 12.3: State whether the study included person-level, institutional-level, or other data linkage across two or more databases. The methods of linkage and methods of linkage quality evaluation should be provided. | *This is reported in the supplementary materials (S6 Appendix)* |
| **Results** | | | | | |
| Participants | 13 | (a) Report the numbers of individuals at each stage of the study (*e.g.*, numbers potentially eligible, examined for eligibility, confirmed eligible, included in the study, completing follow-up, and analysed)  (b) Give reasons for non-participation at each stage.  (c) Consider use of a flow diagram | *This is described in the Results, subsection “Patient Characteristics” and Figure 1* | RECORD 13.1: Describe in detail the selection of the persons included in the study (*i.e.,* study population selection) including filtering based on data quality, data availability and linkage. The selection of included persons can be described in the text and/or by means of the study flow diagram. | *This is described in the Results “Patient Characteristics” and Figure 1* |
| Descriptive data | 14 | (a) Give characteristics of study participants (*e.g.*, demographic, clinical, social) and information on exposures and potential confounders  (b) Indicate the number of participants with missing data for each variable of interest  (c) *Cohort study* - summarise follow-up time (*e.g.*, average and total amount) | *This is done throughout the Results section* |  |  |
| Outcome data | 15 | *Cohort study* - Report numbers of outcome events or summary measures over time  *Case-control study* - Report numbers in each exposure category, or summary measures of exposure  *Cross-sectional study* - Report numbers of outcome events or summary measures | *(Cohort study) This is done early in the Results section, subsection “Outcomes of ART”* |  |  |
| Main results | 16 | (a) Give unadjusted estimates and, if applicable, confounder-adjusted estimates and their precision (e.g., 95% confidence interval). Make clear which confounders were adjusted for and why they were included  (b) Report category boundaries when continuous variables were categorized  (c) If relevant, consider translating estimates of relative risk into absolute risk for a meaningful time period | *This is done throughout the Results section* |  |  |
| Other analyses | 17 | Report other analyses done—e.g., analyses of subgroups and interactions, and sensitivity analyses | *These are mentioned in the manuscript* *Results section, subsection “Outcomes of ART” (final paragraph), as well as in the supplementary materials (S1-S5 Appendix)* |  |  |
| **Discussion** | | | | | |
| Key results | 18 | Summarise key results with reference to study objectives | *This is done at the start of the Discussion (first paragraph)* |  |  |
| Limitations | 19 | Discuss limitations of the study, taking into account sources of potential bias or imprecision. Discuss both direction and magnitude of any potential bias | *This is done in the Discussion, section on limitations (paragraph 10)* | RECORD 19.1: Discuss the implications of using data that were not created or collected to answer the specific research question(s). Include discussion of misclassification bias, unmeasured confounding, missing data, and changing eligibility over time, as they pertain to the study being reported. | *This is done in the Discussion, section on limitations (paragraph 10)* |
| Interpretation | 20 | Give a cautious overall interpretation of results considering objectives, limitations, multiplicity of analyses, results from similar studies, and other relevant evidence | *This is done throughout the Discussion* |  |  |
| Generalisability | 21 | Discuss the generalisability (external validity) of the study results | *Mentioned in the Discussion, section on limitations (paragraph 10)* |  |  |
| **Other Information** | | | | | |
| Funding | 22 | Give the source of funding and the role of the funders for the present study and, if applicable, for the original study on which the present article is based | *Mentioned under “Funding”* |  |  |
| Accessibility of protocol, raw data, and programming code |  | .. |  | RECORD 22.1: Authors should provide information on how to access any supplemental information such as the study protocol, raw data, or programming code. | *Mentioned under “Data availability”* |

References:

1: Benchimol EI, Smeeth L, Guttmann A, Harron K, Moher D, Petersen I, et al. The REporting of studies Conducted using Observational Routinely-collected health Data (RECORD) Statement. PLoS Med. 2015;12(10):1–22..

Checklist is protected under Creative Commons Attribution ([CC BY](http://creativecommons.org/licenses/by/4.0/)) license.
