# Supplementary material for: Virological suppression and clinical management in response to viremia in South African HIV treatment program: A multicenter cohort study
Source: PLoS Med. 2020 Feb 25;17(2):e1003037. doi: 10.1371/journal.pmed.1003037 (PMC7041795; doi:10.1371/journal.pmed.1003037)
Supplement: S2 Appendix — (DOCX) [file pmed.1003037.s002.docx]

## Data Sourcing, Selection and Curation

Data sourcing – Gauteng and North West provinces

Medical record data from 15 clinics in the inner city of Johannesburg (Gauteng province) and 35 clinics in the Dr. Kenneth Kaunda District (North West province) were obtained from the Wits Reproductive Health and HIV Institute (Wits RHI), which supplied technical support as well as monitoring and evaluation of the ART program in these districts from 2004 to 2018. All available medical record data from 1 January 2007 to 1 May 2018 were extracted from Tier.NET.

Laboratory source data were obtained through the National Health Laboratory Services for the 50 clinics for which medical record data was obtained through Wits RHI. All available laboratory source data from 1 January 2007 to 1 May 2016 were extracted.

Data sourcing – Limpopo and Mpumalanga provinces

Medical record data from one clinic in the Sekhukhune district (Limpopo province) and one clinic in the Ehlanzeni district (Mpumalanga province) were obtained from Ndlovu Care Group, which managed a large ART program at these clinics from 2003 to 2018 for the Limpopo site and from 2007 to 2017 for the Mpumalanga site. Data curation by Ndlovu Care Group ensured high quality medical record data, which is challenging to obtain in these settings. All available medical record data from 1 January 2007 to 1 May 2018 were extracted from Tier.NET.

Laboratory source data were not obtained for the clinics in Limpopo and Mpumalanga provinces, as Ndlovu Care Group had previously ensured entry of laboratory source data to the Tier.NET database system when this system was introduced in clinics.

Cohort overlap:

Data from this cohort have been previously reported.[1] This previous work reports on the impact of low-level viremia (51-999 HIV-RNA copies/mL) on outcomes of treatment in this cohort, and incorporated data from 1 January 2007 to 1 May 2016. For the present work, follow-up was expanded until 1 May 2018. Data from several clinics in Johannesburg Region F were not included in the present work due to merging or closure of clinics, resulting in a total of 52 clinics compared to the previous 57 clinics. The total number of patients with a recorded first-line ART treatment episode expanded from 69454 in the previous work to 104719 in the present work. Median follow-up on ART increased from 124 weeks in the previous work to 152 weeks in the present work. Additional data was made available for this analysis, including data on retention, transfer out, and death, allowing for intention-to-treat analysis of virological suppression and more detailed analysis of follow-up after viremia.

Data curation:

Prior to record anonymization, laboratory source data for Johannesburg Region F and Dr. Kenneth Kaunda District were matched to medical record data using a probabilistic matching algorithm. Matching in these districts was performed on a per-clinic basis. The matching algorithm incorporated name, surname, date of birth and sex as matching variables, and accounted for common errors in spelling and omitted data in order to achieve realistic matching. This procedure managed to retrieve crossmatched laboratory source data for 50363 out of 94438 patients. Two separate datasets were created, one containing laboratory source data for each patient and one containing medical record data for each patient.

Deduplication of medical and laboratory records was performed by matching records on name, surname, date of birth, and deduplicating any identical records.

Outliers were removed only in case of improbable continuous data values. CD4 counts above 10.000 cells/uL and dates of birth prior to 01-Jan-1910 were considered outlying and removed.

References:

1: Hermans LE, Moorhouse M, Carmona S, Grobbee DE, Hofstra LM, Richman DD, et al. Effect of HIV-1 low-level viraemia during antiretroviral therapy on treatment outcomes in WHO-guided South African treatment programmes: A multicentre cohort study. Lancet Infect Dis. 2017;18(2):188–97.
