# Supplementary material for: Virological suppression and clinical management in response to viremia in South African HIV treatment program: A multicenter cohort study
Source: PLoS Med. 2020 Feb 25;17(2):e1003037. doi: 10.1371/journal.pmed.1003037 (PMC7041795; doi:10.1371/journal.pmed.1003037)

## Virological Suppression Stratified by Risk Factors For Rebound

Figure 1A-F: Virological suppression over time for patients with and without independent risk factors for viremia.


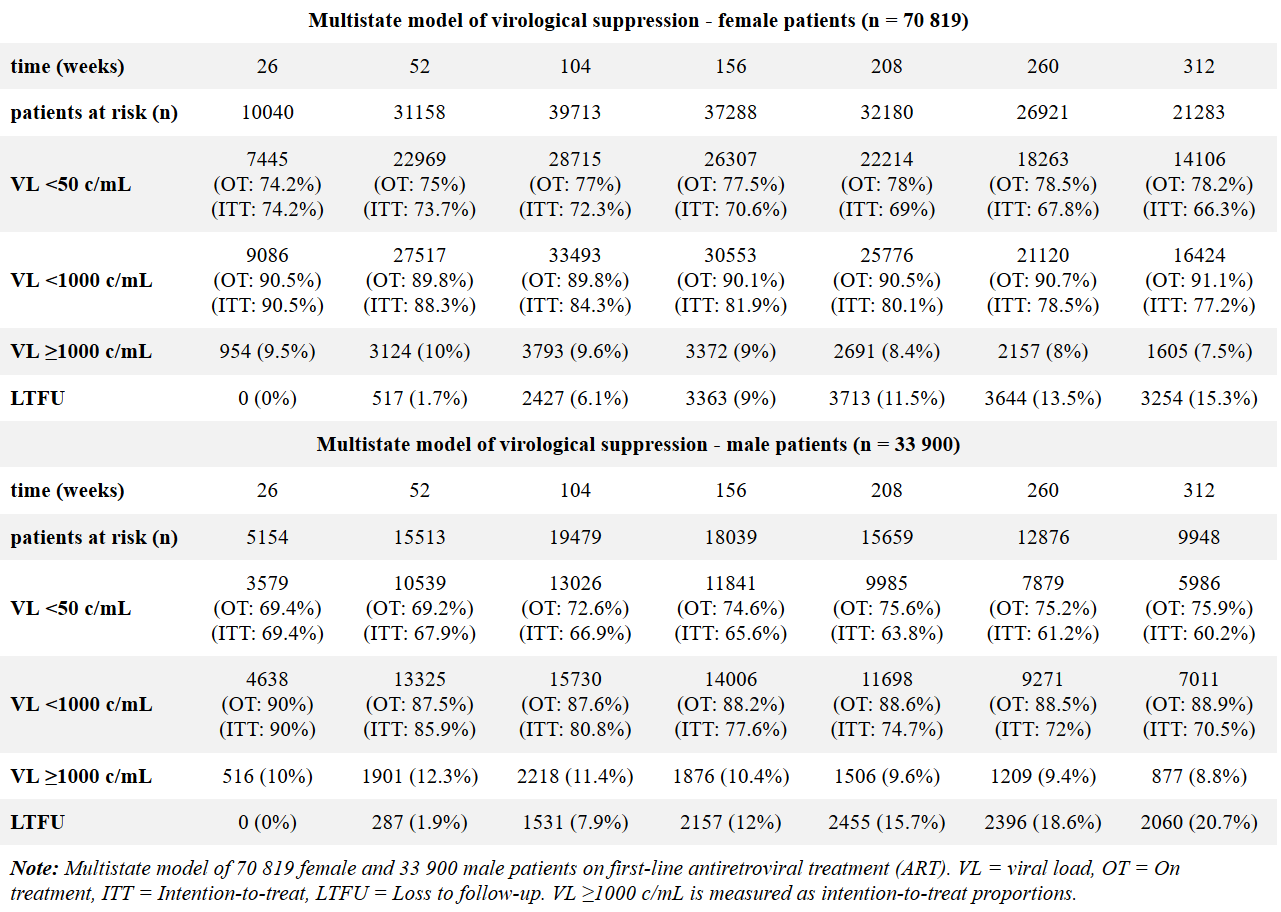


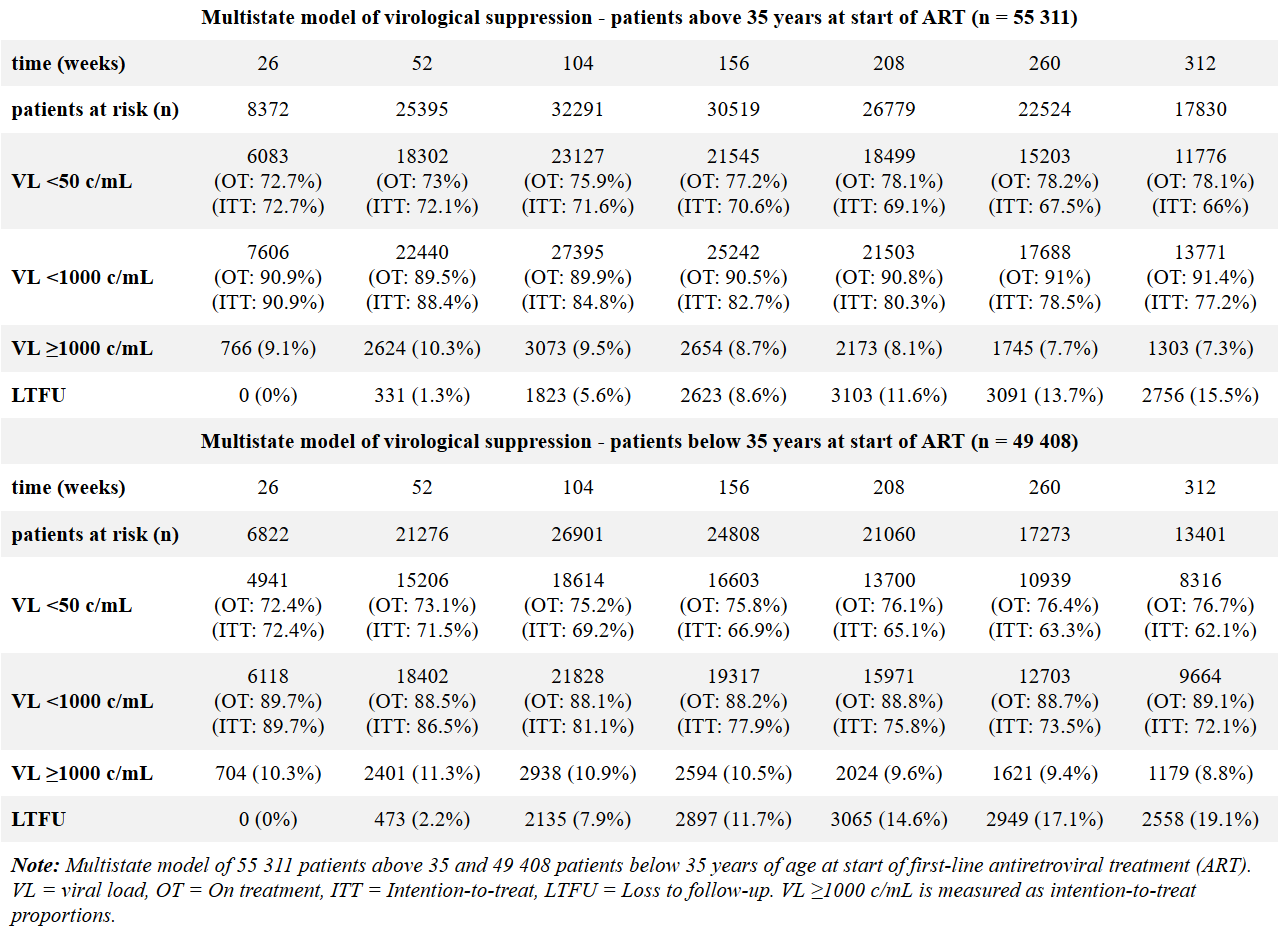


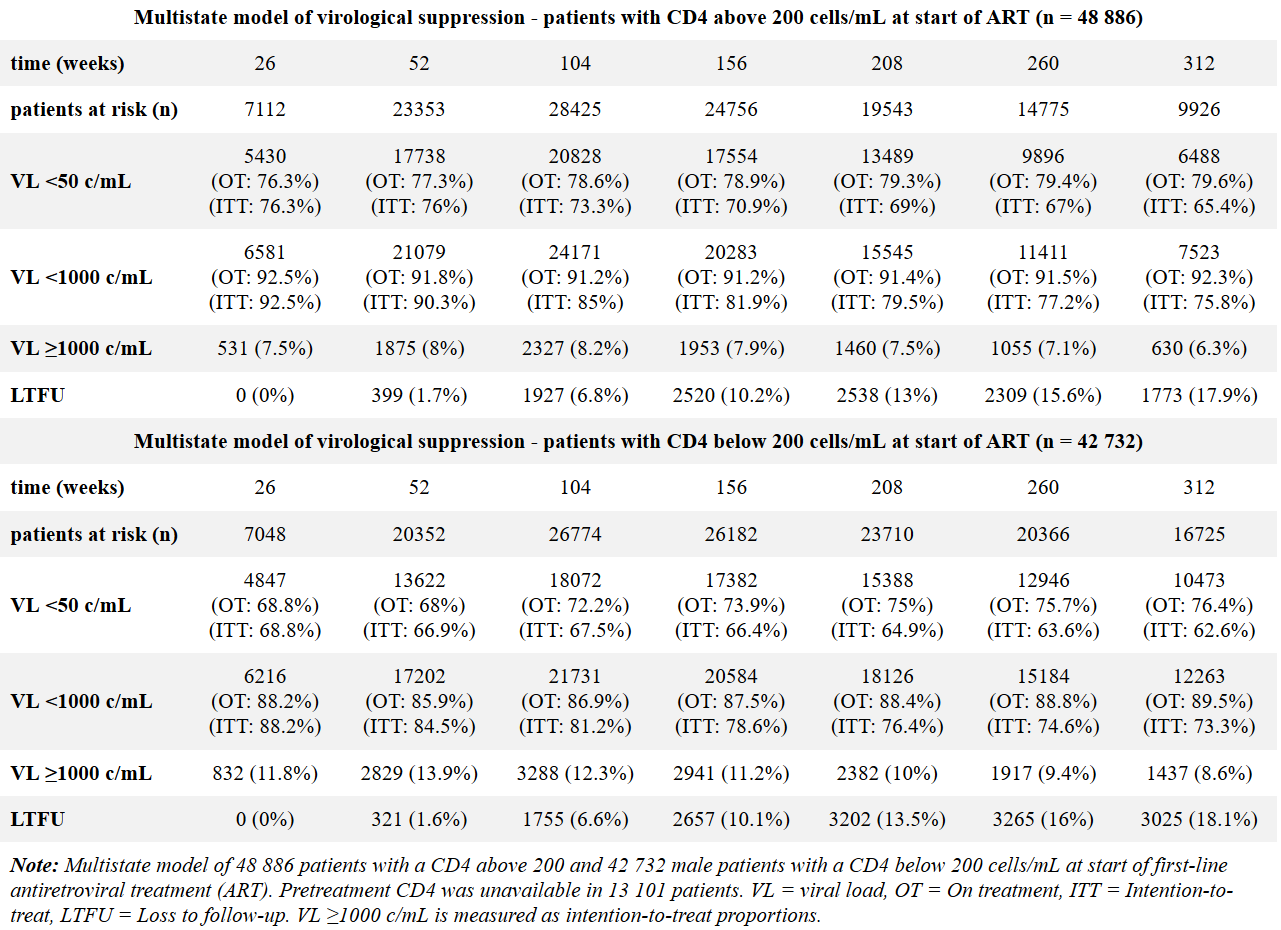

Supplement: S3 Appendix — (DOCX) [file pmed.1003037.s003.docx]
