# Supplementary material for: Virological suppression and clinical management in response to viremia in South African HIV treatment program: A multicenter cohort study
Source: PLoS Med. 2020 Feb 25;17(2):e1003037. doi: 10.1371/journal.pmed.1003037 (PMC7041795; doi:10.1371/journal.pmed.1003037)

## Virological Suppression by Setting

Figure 2A-B: Virological suppression over time for patients in rural and urban-rural mixed settings (Limpopo, Mpumalanga, and North-West province) and urban settings (Gauteng province).


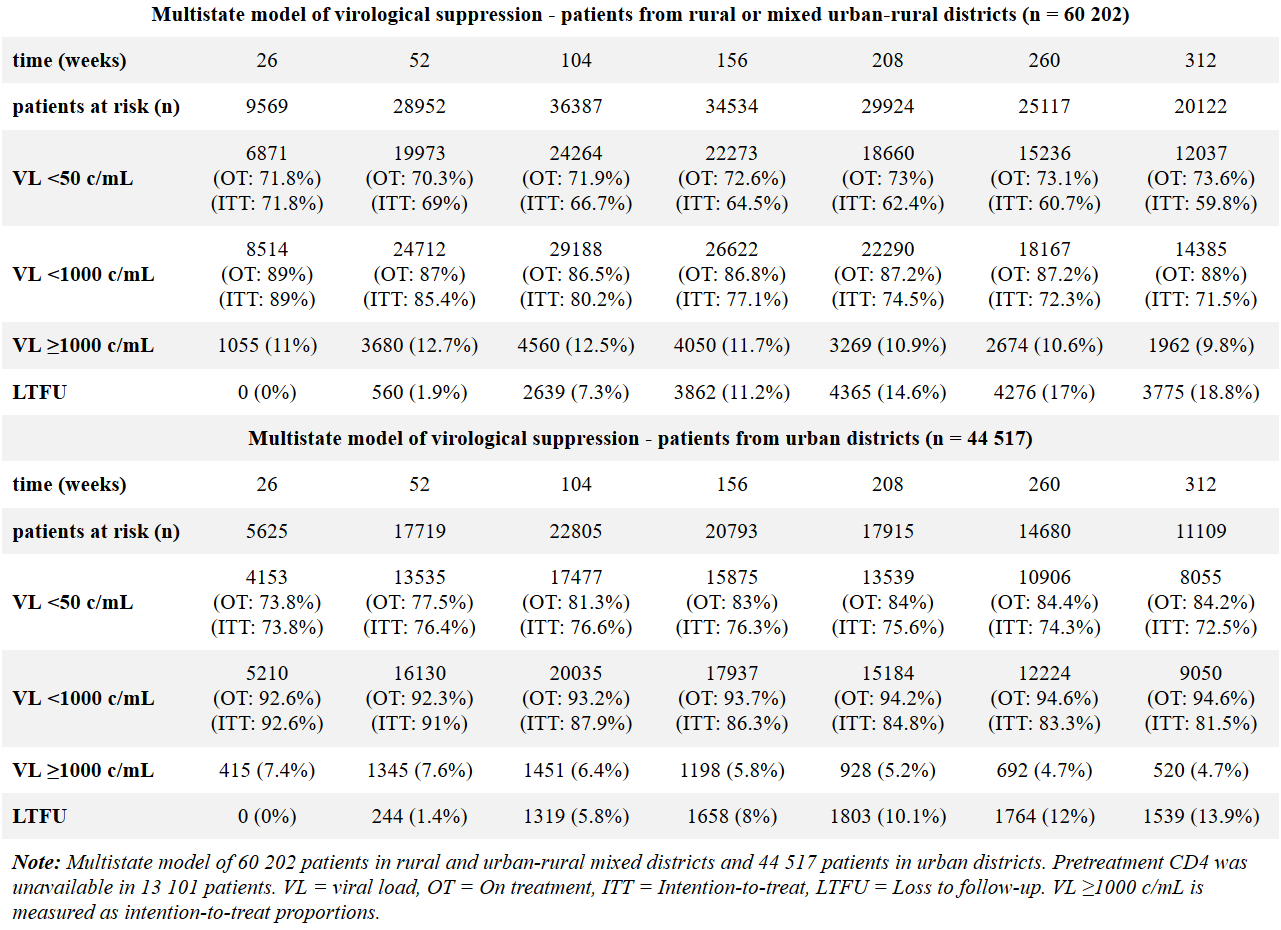

Supplement: S4 Appendix — (DOCX) [file pmed.1003037.s004.docx]
