# Supplementary material for: Virological suppression and clinical management in response to viremia in South African HIV treatment program: A multicenter cohort study
Source: PLoS Med. 2020 Feb 25;17(2):e1003037. doi: 10.1371/journal.pmed.1003037 (PMC7041795; doi:10.1371/journal.pmed.1003037)

## Virological Suppression and Follow-up After Viremia by Data Source

For 50363 out of 94438 patients from the Gauteng and North-West provinces, laboratory source data could be retrieved by probabilistic crossmatching with medical record data. Laboratory source data and medical record data was analysed separately for this group of patients to enable comparison of key study results per data source.

Tabe 1A: Virological suppression according to medical record data and laboratory source data. Comparison between the two sources of data shows that estimates of virological suppression over time are highly comparable between data sources for each exposure group and at each timepoint.


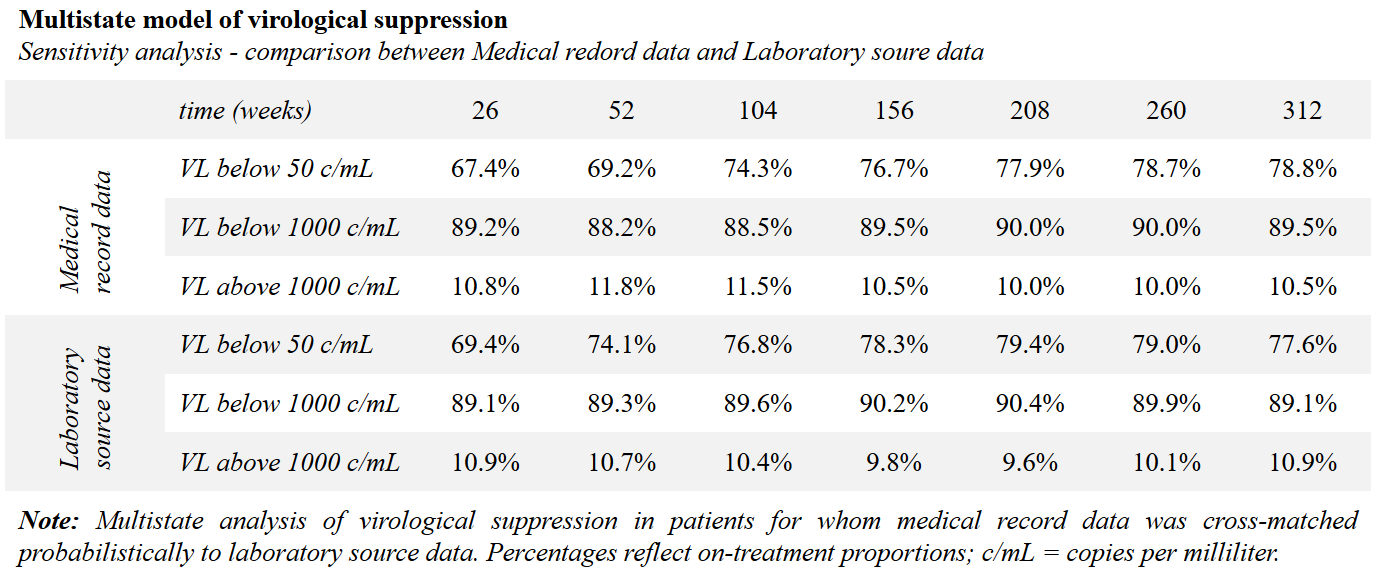


Tabe 1B: Follow-up after detection of viremia according to medical record data and laboratory source data. Comparison between the two sources of data shows that the interval between detection of viral rebound and confirmation of virological failure is 3.6 weeks longer in medical record data compared to laboratory source data, while the interval between detection of viremia and switch is 6.5 weeks shorter in medical record data. Overall, intervals are highly similar between data sources, supporting the findings of the main analysis.


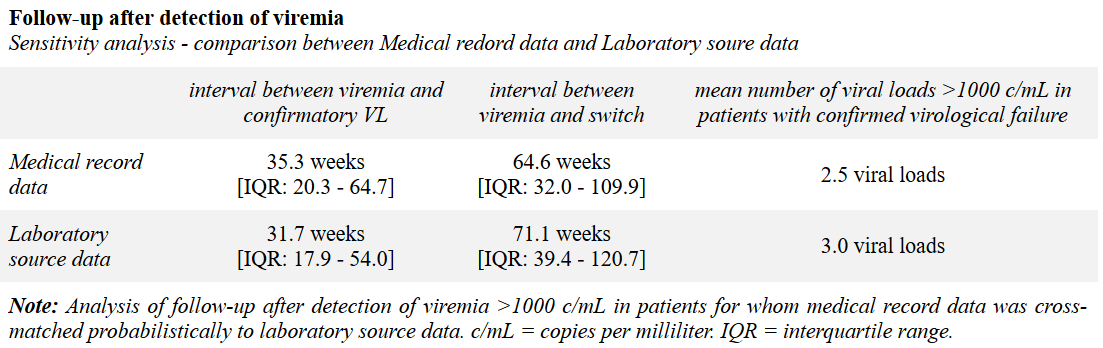

Supplement: S5 Appendix — (DOCX) [file pmed.1003037.s005.docx]
