# Supplementary material for: Virological suppression and clinical management in response to viremia in South African HIV treatment program: A multicenter cohort study
Source: PLoS Med. 2020 Feb 25;17(2):e1003037. doi: 10.1371/journal.pmed.1003037 (PMC7041795; doi:10.1371/journal.pmed.1003037)

## Cox Proportional Hazard Analysis

Table 1: Cox proportional hazards analysis for patients with rebound and subsequent follow-up.


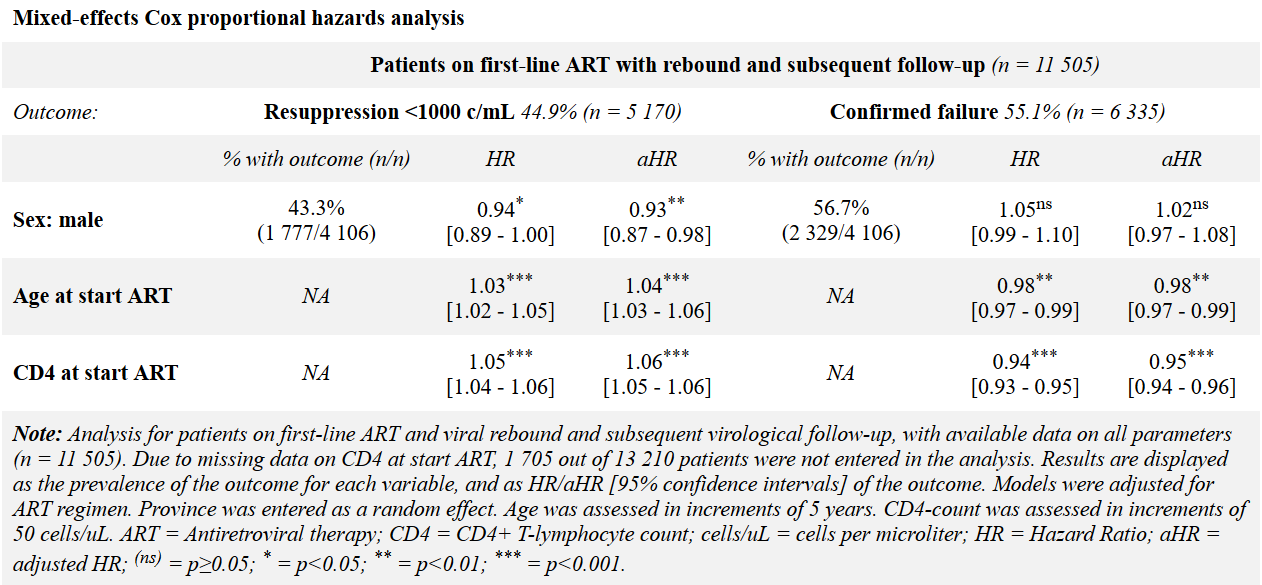


Assumption of proportionality was performed for all Cox proportional hazard models. Inspection of Schoenfeld residuals shows that for certain covariates (Sex, low-level viremia) the assumption of proportionality was not violated in any of the models, whereas for other covariates, particularly age and CD4 count at start of ART, the assumption was violated in most models. The latter covariates are subject to change over time on treatment, and non-proportionality is deemed to be contributable to the fact that these covariates may exert more influence in the early phases of follow-up.

Table 2: Testing of proportional hazard assumption.


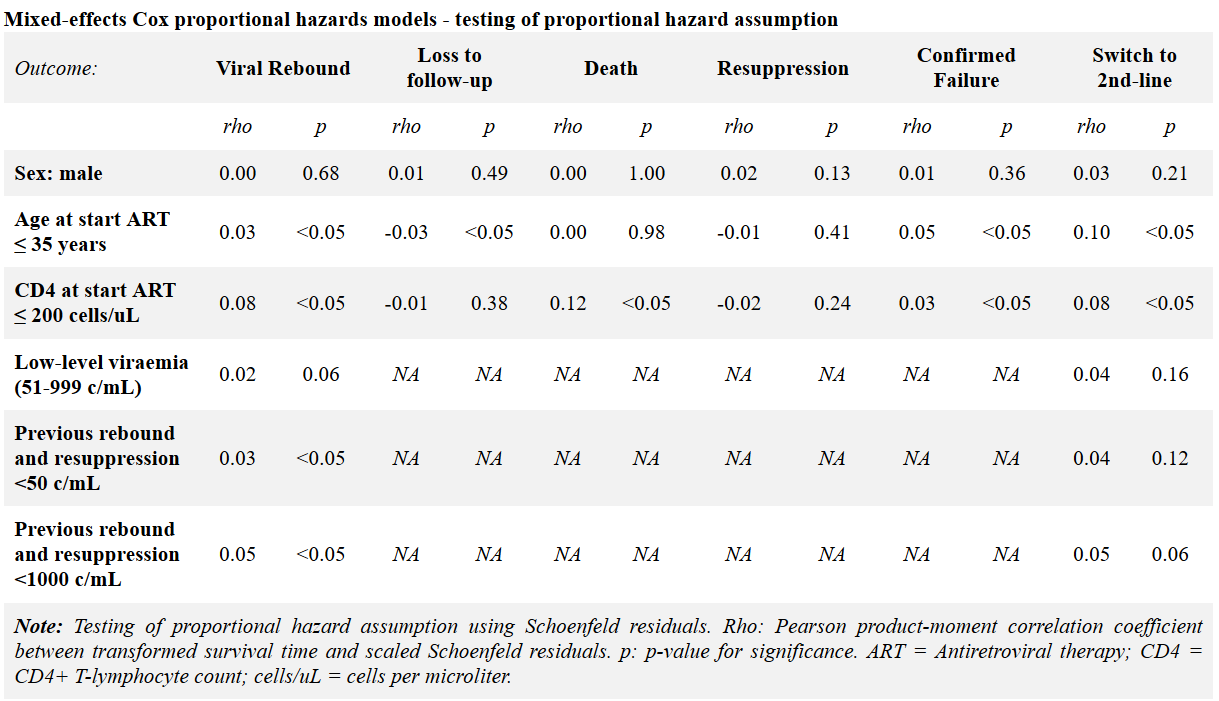


Table 3: Sensitivity analysis comparing all patients with subset of patients with two viral loads or more.


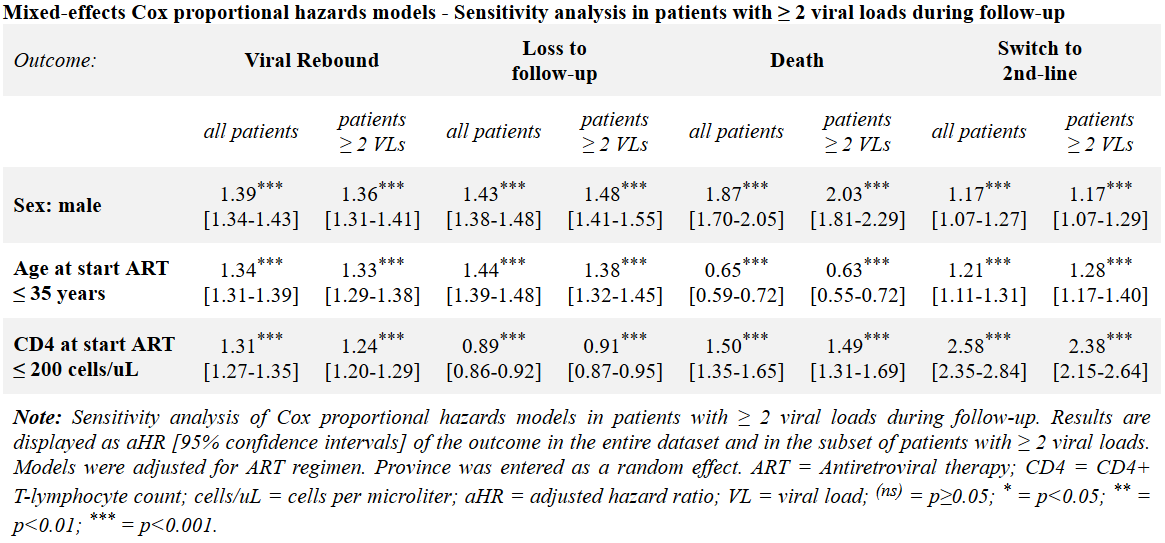

Supplement: S6 Appendix — (DOCX) [file pmed.1003037.s006.docx]
