# Supplementary material for: Virological suppression and clinical management in response to viremia in South African HIV treatment program: A multicenter cohort study
Source: PLoS Med. 2020 Feb 25;17(2):e1003037. doi: 10.1371/journal.pmed.1003037 (PMC7041795; doi:10.1371/journal.pmed.1003037)

## Follow-up after Detection of Viremia by Setting

Follow-up after detection of viremia was evaluated separately for the rural cohort (Limpopo & Mpumalanga provinces), the mixed rural-urban cohort (North-West province) and the urban cohort (Gauteng province).

Table 5: Metrics of follow-up after detection of viremia by setting


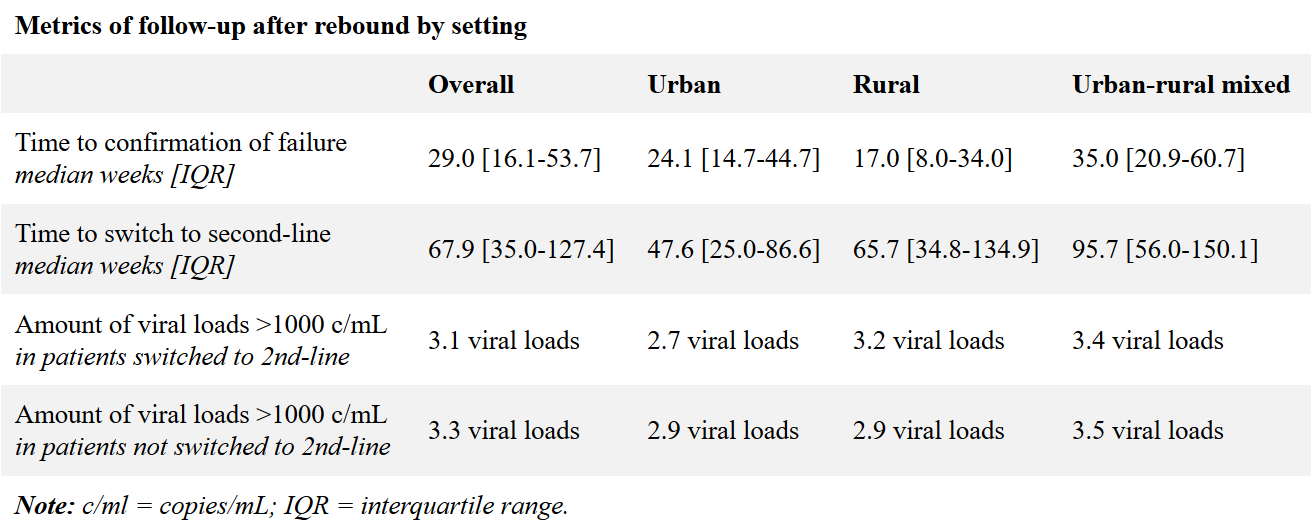

Supplement: S7 Appendix — (DOCX) [file pmed.1003037.s007.docx]
